# The indirect association between psychological well-being and professional commitment through teacher self-efficacy among Turkish language teachers

**DOI:** 10.3389/fpsyg.2026.1901378

**Published:** 2026-10-02

**Authors:** Tuğrul Gökmen Şahin, Emrullah Banaz, Uğur Özbilen

**Affiliations:** 1 Inonu Universitesi, Malatya, Türkiye; 2 Bayburt Universitesi, Bayburt, Türkiye; 3 Hakkari Universitesi, Hakkâri, Türkiye

**Keywords:** professional commitment, psychological well-being, social cognitive theory, teacher self-efficacy, Turkish language teachers

## Abstract

**Introduction:**

This study examined the indirect association between psychological well-being and professional commitment through teacher self-efficacy within the framework of Albert Bandura’s Social Cognitive Theory.

**Methods:**

A correlational research design was employed with a sample of 330 Turkish language teachers working in public schools in Türkiye. Data were collected using the Teacher Self-Efficacy Scale, the Psychological Well-Being Scale, and the Teachers’ Professional Commitment Scale. Pearson correlation and regression-based indirect-effect analyses were conducted using PROCESS macro Model 4 developed by Andrew F. Hayes.

**Results:**

Psychological well-being was positively and significantly associated with both teacher self-efficacy and professional commitment. Teacher self-efficacy was also positively and significantly associated with professional commitment. Furthermore, the indirect-effect analysis indicated a statistically significant indirect association between psychological well-being and professional commitment through teacher self-efficacy. The proposed model explained approximately 30% of the variance in professional commitment.

**Discussion:**

The findings highlight the relevance of teachers’ psychological well-being and self-efficacy to professional commitment and provide context-specific evidence from Turkish language teachers working in the Turkish educational context.

## 1 Introduction

Understanding the relationships between teachers’ psychological resources and their professional attitudes has become an increasingly important focus of educational research. Among these resources, psychological well-being has been consistently associated with teachers’ motivation, resilience, and commitment to their profession. According to Social Cognitive Theory (Bandura, 2001), individuals’ beliefs regarding their own capabilities constitute an important component of the relationship between personal psychological resources and professional functioning. Within educational settings, teacher self-efficacy reflects teachers’ beliefs in their ability to effectively manage instructional processes and facilitate student learning (Skaalvik and Skaalvik, 2007). From this theoretical perspective, teacher self-efficacy may be relevant to understanding the association between psychological well-being and professional commitment. Accordingly, the present study examines whether psychological well-being is indirectly associated with professional commitment through teacher self-efficacy.

Previous research has consistently demonstrated that teacher self-efficacy is positively associated with desirable professional outcomes, including job satisfaction, work engagement, and professional commitment. Self-efficacy beliefs have also been associated with lower burnout and reduced intentions to leave the profession prematurely (Capone et al., 2022; Ma et al., 2022; Shu, 2022; Xi-yun et al., 2022). Psychological well-being has likewise been positively associated with teachers’ self-efficacy beliefs (Fırat, 2025). Taken together, these findings provide a theoretical and empirical basis for examining whether teacher self-efficacy statistically accounts for part of the association between psychological well-being and professional commitment. However, given the cross-sectional nature of the present study, the proposed indirect relationship is examined as a statistical association rather than as evidence of a temporal or causal mechanism (Alibakhshi et al., 2020; Dicke et al., 2018).

Against this backdrop, the present study aims to examine the associations among psychological well-being, teacher self-efficacy, and professional commitment among Turkish language teachers working in Türkiye. The Turkish educational context provides a distinctive setting characterized by particular educational policies, working conditions, and socio-cultural dynamics. Examining these relationships within this context may contribute to the broader literature by providing context-specific evidence regarding the applicability of existing theoretical assumptions. Accordingly, the following hypotheses were developed.

## 2 Conceptual framework

### 2.1 Psychological well-being and teacher self-efficacy

Self-efficacy beliefs refer to individuals’ judgments regarding their capabilities to organize and execute the courses of action required to attain desired outcomes (Bandura, 1997). Within educational research, teacher self-efficacy is conceptualized as a multidimensional construct encompassing teachers’ perceptions of their competence in classroom management, instructional strategies, and fostering student engagement (Tschannen-Moran and Hoy, 2001). Teachers with higher levels of self-efficacy are more likely to employ innovative instructional practices, implement strategies that support student achievement, and demonstrate greater resilience in coping with professional challenges (Klassen and Chiu, 2010). Accordingly, teacher self-efficacy represents an important psychological construct for understanding the relationships between teachers’ psychological resources and their professional attitudes.

According to Social Cognitive Theory (Bandura, 2001), self-efficacy beliefs constitute one of the fundamental psychological mechanisms shaping individuals’ motivation, the effort they invest in tasks, their persistence when confronted with difficulties, and ultimately their performance. In professions such as teaching, which require complex cognitive, emotional, and interpersonal competencies, self-efficacy beliefs are considered particularly influential in determining instructional behaviors and overall teaching effectiveness (Çolak, 2025; Li et al., 2025; Ma et al., 2022; Xi-yun et al., 2022). From a Social Cognitive Theory perspective, cognitive, affective, and environmental factors continuously interact through the principle of reciprocal determinism, while self-regulatory processes shape both instructional practices and individuals’ psychological well-being (Chen et al., 2024; Çetin et al., 2024; Wang and Pan, 2023). Within this framework, teachers’ psychological resources and self-efficacy beliefs can be understood as interrelated components of professional functioning and instructional effectiveness.

Teacher self-efficacy is widely recognized as a key psychological resource that contributes to improved teaching performance. Teachers with stronger self-efficacy beliefs tend to demonstrate greater persistence in instructional efforts, employ more adaptive coping strategies when facing classroom challenges, and exhibit more effective teaching practices. Consequently, higher levels of teacher self-efficacy have been associated with more positive student outcomes, greater perceived instructional effectiveness, and increased professional satisfaction (Chen et al., 2024; Wang and Pan, 2023; Zhi et al., 2023). Empirical evidence grounded in Social Cognitive Theory further indicates that self-efficacy is strongly associated with various indicators of teacher performance and is linked, both directly and indirectly, to well-being-related outcomes such as work engagement, job satisfaction, and reduced burnout (Heng and Chu, 2023; Shao, 2023; Xi-yun et al., 2022; Zhang et al., 2023).

Based on the theoretical and empirical evidence presented above, psychological well-being is expected to be positively and significantly associated with teacher self-efficacy among teachers in Türkiye. Accordingly, the following hypothesis was formulated:

*H1*: Psychological well-being is positively and significantly associated with teacher self-efficacy.

### 2.2 Psychological well-being and professional commitment

Psychological well-being is a multidimensional construct encompassing individuals’ perceptions of subjective health, sustained happiness, and positive psychological functioning. Although psychological well-being may encompass multiple dimensions of positive functioning, the present study operationalized it as an overall psychological well-being construct. This approach is consistent with the unidimensional structure of the Psychological Well-Being Scale used in the study, in which the eight items are specified to load on a single factor. Accordingly, the analyses focus on teachers’ overall level of psychological well-being rather than on separate dimensions of well-being. Within educational settings, psychological well-being has been associated with teachers’ professional quality of life, motivation, and performance-related outcomes (Mérida-López et al., 2017; Yue et al., 2016; Zhang, 2022). Accordingly, psychological well-being represents an important psychological construct for understanding teachers’ professional experiences and attitudes.

Previous research has reported significant associations between teachers’ psychological well-being and a range of individual and professional characteristics, including self-efficacy beliefs, coping with workload demands, and attitudes toward administrative processes (Yue et al., 2016; Zhang, 2022). In addition, teachers’ psychological well-being has been examined in relation to students’ motivation and learning-related outcomes (Hamu et al., 2022; Han, 2022). Taken together, these findings indicate that psychological well-being is meaningfully associated with multiple aspects of teachers’ professional functioning, while the direction and causal nature of these relationships depend on the designs employed in individual studies.

Professional commitment can be conceptualized as a multidimensional construct reflecting different aspects of individuals’ attachment to and evaluation of their profession. In the present study, professional commitment encompasses Professional Satisfaction, Social Gain, and Professional Career. Although these dimensions represent distinguishable aspects of professional commitment, the present study focuses on the broader construct because its primary hypotheses concern teachers’ overall professional commitment rather than dimension-specific associations. Accordingly, professional commitment is examined at the aggregate level in the primary analyses. Contemporary theoretical and empirical evidence indicates a positive association between psychological well-being and professional commitment. Individuals reporting higher levels of psychological well-being also tend to perceive their work as more meaningful and purposeful and to report greater job satisfaction and professional attachment (Cignitas et al., 2022; Heidari et al., 2022; Kundi et al., 2020; Lee and Kim, 2023b). These findings suggest that psychological well-being is meaningfully related to professional attitudes, including satisfaction and commitment. Examining this association among Turkish language teachers in Türkiye provides an opportunity to extend existing evidence within a distinctive educational and cultural context.

Previous research further suggests that the association between psychological well-being and professional commitment may involve other psychological and work-related variables. For example, job satisfaction has been examined as an intervening variable in the relationship between psychological well-being and work-related commitment (Lee and Kim, 2023a; Lee and Kim, 2023b). Psychological resources such as emotional energy, goal-directed professional motivation, and perceived meaning in work have likewise been associated with stronger professional and organizational attachment (Heidari et al., 2022; Krajcsák, 2018; Kundi et al., 2020; Mahmood et al., 2023). Collectively, this literature supports examining indirect statistical relationships among psychological resources and professional commitment while recognizing that the direction and temporal ordering of these relationships cannot be established from cross-sectional evidence alone.

Based on the theoretical framework and empirical evidence presented above, psychological well-being is expected to be positively and significantly associated with professional commitment among teachers in Türkiye. Accordingly, the following hypothesis was formulated:

*H2*: Psychological well-being is positively and significantly associated with teachers’ professional commitment.

### 2.3 Teacher self-efficacy and professional commitment

The relationship between teacher self-efficacy and professional commitment has received considerable attention in the educational literature, given its central role in shaping teachers’ motivation, job satisfaction, burnout experiences, organizational behaviors, and career sustainability (Ahn and Bowers, 2024; Leijen et al., 2024; Pan, 2023; Polatcan et al., 2019; Skaalvik and Skaalvik, 2007). In this regard, a substantial body of research has demonstrated the influential role of teacher self-efficacy in determining both teacher- and student-related outcomes. Teacher self-efficacy, in particular, has been identified as one of the key factors influencing students’ academic achievement and motivation (Klassen and Tze, 2014). Moreover, teachers with stronger self-efficacy beliefs tend to report higher levels of job satisfaction, which in turn strengthens their intention to remain in the profession and reduces the likelihood of leaving teaching prematurely (Wang and Zhang, 2021; Zakariya, 2020). Similarly, empirical evidence indicates that teachers who possess higher levels of self-efficacy demonstrate stronger commitment to their profession (Mokhtar et al., 2023; Yurtseven and Dulay, 2022). Supporting this view, Zee and Koomen (2016) concluded that teachers with stronger self-efficacy beliefs experience lower levels of occupational stress and higher levels of job satisfaction. Taken together, these findings suggest that teacher self-efficacy is a multidimensional and dynamic construct that is both influenced by and influential in relation to a wide range of individual and organizational factors.

Teacher self-efficacy may also be relevant to understanding the relationship between teachers’ psychological resources and their professional attitudes. Social Cognitive Theory (Bandura, 2001) emphasizes the importance of efficacy beliefs in individuals’ motivation, persistence, and professional functioning. Within this theoretical framework, teachers reporting greater psychological well-being may also report stronger beliefs in their professional capabilities, while stronger self-efficacy beliefs may coexist with greater commitment to the teaching profession. These theoretically expected relationships provide a basis for examining teacher self-efficacy within the association between psychological well-being and professional commitment.

Teachers who possess strong beliefs in their ability to effectively manage instructional processes are more likely to perceive teaching as a meaningful, rewarding, and sustainable career. Consequently, teacher self-efficacy may be regarded as an important psychological resource that is positively associated with teachers’ professional commitment. Based on this theoretical rationale and empirical evidence, the following hypothesis was formulated:

*H3*: Teacher self-efficacy is positively and significantly associated with teachers’ professional commitment.

### 2.4 The indirect association through teacher self-efficacy

Social Cognitive Theory (Bandura, 2001) provides a theoretical basis for considering teacher self-efficacy within the relationship between psychological well-being and professional commitment. Psychological well-being has been positively associated with self-efficacy-related beliefs, while teacher self-efficacy has been positively associated with professional attitudes, including professional and organizational commitment. Taken together, these relationships provide a rationale for examining whether psychological well-being is indirectly associated with professional commitment through teacher self-efficacy.

A review of previous studies (Jadoon et al., 2023; Kong, 2021; Palmer, 2011; Setyaningsih and Sunaryo, 2021; Shimony et al., 2022), together with the literature discussed above, indicates that psychological well-being, teacher self-efficacy, and professional commitment have frequently been examined in separate or direct relationships. Comparatively less attention has been given to whether teacher self-efficacy statistically accounts for part of the association between psychological well-being and professional commitment. The present study addresses this issue by examining the indirect association between psychological well-being and professional commitment through teacher self-efficacy. Examining this pattern among Turkish language teachers also provides context-specific evidence from the Turkish educational system. Related context-specific evidence has been reported by Özbilen et al. (2024), who examined teacher self-efficacy, career sustainability, job satisfaction, and subjective well-being among Turkish language teachers. Their findings showed that career sustainability played a significant mediating role in the relationships between teacher self-efficacy and subjective well-being and between teacher self-efficacy and job satisfaction. Although their model addressed a different mediating mechanism and different outcomes, the study provides relevant empirical evidence that self-efficacy and well-being-related constructs are interconnected within the same professional population examined in the present study.

Empirical research provides support for the individual associations underlying this proposed model. Previous studies have reported positive associations between teacher self-efficacy and psychological well-being (Cho et al., 2021), as well as between self-efficacy and teacher commitment (Ford et al., 2019; Li et al., 2024; Sanford, 2017). Other studies have similarly identified indirect statistical relationships involving psychological well-being and work-related commitment (Altınay and Bicentürk, 2023; Gan and Cheng, 2021). However, empirical evidence concerning the specific indirect association examined in the present study remains limited, particularly among Turkish language teachers.

Accordingly, the present study examines whether psychological well-being is indirectly associated with professional commitment through teacher self-efficacy. This model is theoretically informed by Social Cognitive Theory but is tested using cross-sectional data. Therefore, any statistically significant indirect association is interpreted as evidence consistent with the proposed theoretical model rather than as evidence that teacher self-efficacy constitutes a temporal or causal mechanism.

Based on the theoretical framework and empirical evidence reviewed above, the following hypothesis was formulated:

*H4*: Psychological well-being is indirectly and significantly associated with professional commitment through teacher self-efficacy.

## 3 Research design

This study employed a correlational research design. Correlational research is a quantitative approach that enables researchers to describe individuals’ tendencies, attitudes, and perceptions based on data collected from a sample drawn from a target population, while also examining the nature of the relationships among variables (Creswell, 2016). The primary purpose of correlational research is to determine whether variables co-vary and, if so, to identify the direction and strength of these associations. Previous methodological literature has emphasized the effectiveness of correlational designs in examining complex multivariate structures and uncovering patterns of relationships among variables (Büyüköztürk, 2011; Fraenkel et al., 2012).

Correlational research also permits the examination of indirect statistical associations among multiple variables. In the present study, this design enabled the simultaneous examination of the direct associations among psychological well-being, teacher self-efficacy, and professional commitment, as well as the indirect association between psychological well-being and professional commitment through teacher self-efficacy (Field, 2018; Tabachnick and Fidell, 2013). Because the data were collected at a single time point, the indirect-effect analysis was used to evaluate whether the observed pattern of associations was consistent with the theoretically proposed model rather than to establish a causal or temporal mediation process.

### 3.1 Participants

The study sample consisted of 330 Turkish language teachers employed in public schools affiliated with the Ministry of National Education in Türkiye. Participants were selected using convenience sampling based on voluntary participation and accessibility considerations. Participants’ province or geographical region of employment was not recorded during data collection; therefore, the geographical distribution of the sample across Türkiye cannot be determined. Although all participants were Turkish language teachers employed in public schools affiliated with the Ministry of National Education and thus worked within the same national institutional framework, this common institutional context should not be interpreted as indicating uniform working conditions across geographical regions.

Among the teachers, 60.3% were male (*n* = 199) and 39.7% were female (*n* = 131). The sample represented a broad range of professional experience. Specifically, 43.0% of the teachers had between 1 and 5 years of teaching experience (*n* = 142), whereas 33.0% had 15 years or more of professional experience (*n* = 109). Regarding educational attainment, most participants held a bachelor’s degree (77.3%, *n* = 255), while 22.7% (*n* = 75) had completed postgraduate education.

The target population of the study comprised Turkish language teachers employed in public schools affiliated with the Ministry of National Education in Türkiye. However, because participants were recruited through convenience sampling and voluntary participation via online communication channels, the sample should not be considered statistically representative of this target population. The demographic characteristics reported in Table 1 are therefore provided to describe the composition of the sample rather than to establish population representativeness. Accordingly, the findings should be interpreted as pertaining to the participating teachers, and their generalizability to the broader population of Turkish language teachers should be approached with caution.

**Table 1:** Participants’ demographics.

Characteristic		*n*	%
Gender	Male	199	60.3
	Female	131	39.7
Professional seniority (years)	1–5	142	43.0
	6–10	37	11.2
	11–15	42	12.7
	> 15	109	33.0
Education	Undergraduate	255	77.3
	Postgraduate	75	22.7

An *a priori* power analysis was not conducted before data collection. Therefore, we did not retrospectively characterize the observed sample size as having been prospectively determined through power analysis. Nevertheless, the adequacy of the final sample (*N* = 330) was evaluated with reference to methodological evidence concerning statistical power in mediation analysis. Fritz and MacKinnon (2007) demonstrated that the sample size required to achieve.80 power for detecting an indirect effect varies substantially as a function of the magnitudes of the *a* and *b* paths and the statistical procedure used to test mediation. Given that the present study employed a bootstrap-based test of the indirect effect, the sample of 330 provides a reasonable basis for detecting nontrivial indirect associations; however, it may be insufficient for very small indirect effects. Accordingly, the absence of a prospectively conducted power analysis is acknowledged as a methodological limitation.

### 3.2 Data collection tools

#### 3.2.1 Teacher Self-Efficacy Scale

Teacher self-efficacy was assessed using the Teacher Self-Efficacy Scale originally developed by Friedman and Kass (2002) and adapted into Turkish by Arslan (2022). Following the adaptation study, four items (Items 4, 7, 10, and 14) were removed based on confirmatory factor analysis results, resulting in a unidimensional 10-item scale. Responses are rated on a six-point Likert scale ranging from 1 (*strongly disagree*) to 6 (*strongly agree*).

The adaptation study supported the single-factor structure of the scale and demonstrated acceptable model fit indices (CFI = 0.952, TLI = 0.939, NFI = 0.936, IFI = 0.953, SRMR = 0.080, GFI = 0.978). Item factor loadings exceeded the recommended threshold of 0.40. In the present study, the scale demonstrated good internal consistency, with a Cronbach’s alpha coefficient of.83.

In the current sample, confirmatory factor analysis was conducted to examine the proposed unidimensional structure of the scale. The initial model showed inadequate fit, χ^2^(35) = 193.26, CFI = 0.881, TLI = 0.847, RMSEA = 0.117, and SRMR = 0.073. Inspection of localized areas of misfit, together with consideration of item content, indicated shared residual variance between Items 7 and 8 and between Items 5 and 10. Items 7 and 8 both concern teachers’ responses to interpersonal or organizational difficulties at school, particularly access to support and withdrawal when difficulties arise. Items 5 and 10 both concern teachers’ perceived functioning within the school environment, although one emphasizes perceived capacity to contribute to problem solving and the other emphasizes positive collegial relationships. These content similarities may account for item-specific covariance beyond the general teacher self-efficacy factor. Accordingly, the residual covariances between these item pairs were freely estimated. The respecified model demonstrated substantially improved and acceptable fit, χ^2^(33) = 89.52, CFI = 0.958, TLI = 0.942, RMSEA = 0.072, and SRMR = 0.052. These results provided support for the unidimensional structure of the Teacher Self-Efficacy Scale in the current sample.

#### 3.2.2 Psychological Well-Being Scale

Psychological well-being was measured using the short form of the Psychological Well-Being Scale developed by Diener et al. (2010), based on the original version proposed by Diener et al. (2009). The Turkish adaptation of the scale was conducted by Telef (2013). The scale consists of eight items loading on a single factor and is rated on a seven-point Likert scale ranging from 1 (*strongly disagree*) to 7 (*strongly agree*).

Results from the Turkish adaptation study indicated that the scale explained 42% of the total variance, with factor loadings ranging from 0.54 to 0.76. Confirmatory factor analysis supported the unidimensional structure, yielding acceptable fit indices (RMSEA = 0.08, SRMR = 0.04, GFI = 0.96, NFI = 0.94, RFI = 0.92, CFI = 0.95, IFI = 0.95). Telef (2013) reported a Cronbach’s alpha coefficient of 0.80. In the present study, the internal consistency coefficient was 0.85. Higher scores indicate higher levels of psychological well-being.

In the current sample, confirmatory factor analysis was conducted to examine the proposed unidimensional structure of the Psychological Well-Being Scale. The initial model showed inadequate fit, χ^2^(20) = 121.35, CFI = 0.887, TLI = 0.842, RMSEA = 0.124, and SRMR = 0.068. Inspection of localized areas of misfit, together with consideration of item content, indicated shared residual variance between Items 6 and 7, Items 4 and 5, Items 1 and 7, and Items 5 and 8. Items 6 and 7 both reflect a broadly positive orientation toward one’s life, with the former emphasizing a positive evaluation of one’s present life and the latter optimism about the future. Items 1 and 7 similarly share a future-oriented positive perspective, linking a sense of purpose and meaning with optimism. Items 4 and 5 both concern positive functioning and perceived capability, reflected, respectively, in contributing to others’ well-being and feeling competent in personally important activities. Finally, Items 5 and 8 both involve positive evaluations of personal worth and functioning, expressed through perceived competence and perceived respect from others. These content similarities provide a substantive basis for the observed item-specific covariance. Accordingly, the residual covariances between these item pairs were freely estimated. The respecified model demonstrated substantially improved and acceptable fit, χ^2^(16) = 44.62, CFI = 0.968, TLI = 0.944, RMSEA = 0.074, and SRMR = 0.044. These results provided support for the unidimensional structure of the Psychological Well-Being Scale in the current sample.

#### 3.2.3 Teachers’ Professional Commitment Scale

Professional commitment was measured using the Teachers’ Professional Commitment Scale developed by Ergen (2016) for teachers in Türkiye. The development of the instrument was informed by existing measures of professional commitment and qualitative input obtained from 25 classroom teachers, who were asked to describe the characteristics of a teacher committed to the profession. Based on the literature and teachers’ responses, an initial 41-item, five-point Likert-type item pool was generated and subsequently refined to 36 items following content and language review.

For the factor-analytic stage of scale development, the 36-item form was administered to 457 teachers working in the central districts of Malatya, of whom 361 provided usable responses for analysis. Exploratory factor analysis resulted in a 14-item, three-factor structure comprising Professional Satisfaction, Social Gain, and Professional Career, which accounted for 59.94% of the total variance. Factor loadings ranged from 0.56 to 0.79 for Professional Satisfaction, 0.53 to 0.80 for Social Gain, and 0.78 to 0.84 for Professional Career. Confirmatory factor analysis subsequently supported the three-factor structure (χ^2^/df = 1.95, RMSEA = 0.071, RMR = 0.073, NFI = 0.93, NNFI = 0.94, CFI = 0.95, IFI = 0.95, RFI = 0.91, GFI = 0.90, AGFI = 0.88). The original study also reported an internal consistency coefficient of 0.84 and a split-half reliability coefficient of 0.82. In the present study, Cronbach’s alpha was 0.82, indicating satisfactory internal consistency.

In the current sample, confirmatory factor analysis was conducted to examine the original three-factor structure of the Teachers’ Professional Commitment Scale. The initial model showed inadequate fit, χ^2^(74) = 311.08, CFI = 0.882, TLI = 0.854, RMSEA = 0.099, and SRMR = 0.074. Inspection of localized areas of misfit indicated substantial shared residual variance between Items 10 and 11, both of which assess closely related aspects of favorable working conditions and opportunities for rest within the Social Gain dimension. Accordingly, the residual covariance between these two items was freely estimated. The respecified three-factor model demonstrated substantially improved and acceptable fit, χ^2^(73) = 160.89, CFI = 0.956, TLI = 0.945, RMSEA = 0.060, and SRMR = 0.048. Standardized factor loadings ranged from 0.46 to 0.84.

Because the indirect-effect analysis operationalized professional commitment as an overall construct, the appropriateness of using an aggregate score was also examined. The three first-order dimensions were moderately to strongly correlated (r = 0.54–0.71), suggesting substantial shared variance. A higher-order representation further indicated strong standardized loadings of Professional Satisfaction (0.94), Social Gain (0.75), and Professional Career (0.71) on an overarching professional commitment factor. Because a higher-order model with only three first-order factors is just-identified at the higher-order level, these results were not interpreted as evidence of superior model fit; rather, they provide supporting evidence that the three dimensions reflect a common broader construct. On this basis, and because the present study focused on overall professional commitment rather than dimension-specific associations, the total scale score was retained for the indirect-effect analysis. Accordingly, the aggregate professional commitment score, rather than the three subscale scores separately, was used as the outcome variable in the primary hypothesis tests, consistent with the study’s focus on teachers’ overall professional commitment.

### 3.3 Data analysis

The relationships among psychological well-being, teacher self-efficacy, and professional commitment were examined within a regression-based indirect-effect framework. Prior to the main analyses, the dataset was screened for missing data, outliers, and univariate normality assumptions using skewness and kurtosis values (Çokluk et al., 2021). The initial dataset consisted of 337 participants; however, data from seven participants were excluded due to invalid or outlying responses. Consequently, all analyses were conducted using data obtained from 330 participants. To evaluate the measurement structure of the instruments in the current sample, confirmatory factor analyses (CFAs) were conducted for the Psychological Well-Being Scale, Teacher Self-Efficacy Scale, and Teachers’ Professional Commitment Scale prior to the main analyses. Because all study variables were assessed using self-report measures collected within the same survey administration, potential common method variance was examined using Harman’s single-factor test (Podsakoff et al., 2003). All 32 measurement items were entered simultaneously into an unrotated exploratory factor analysis. The first factor accounted for 30.20% of the total variance, which was below the commonly used 50% criterion. Thus, the test did not indicate that a single common factor accounted for the majority of variance in the observed measures. Nevertheless, given the limitations of Harman’s single-factor test, this result was interpreted as a preliminary diagnostic rather than definitive evidence that common method bias was absent.

Descriptive statistics, including frequencies (n), percentages (%), means (M), and standard deviations (SD), were calculated to summarize the data. Pearson correlation analyses were subsequently performed to examine the bivariate associations among the study variables. To examine the hypothesized indirect association between psychological well-being and professional commitment through teacher self-efficacy, a regression-based indirect-effect analysis was conducted. Before testing the proposed model, multicollinearity diagnostics were examined. The obtained tolerance (0.76) and variance inflation factor (VIF = 1.31) values indicated that multicollinearity was not a concern (Field, 2018; Tabachnick and Fidell, 2013). The indirect-effect analysis was performed using PROCESS Macro Version 4.0 developed by Hayes (2022), with Model 4 specified for the proposed model. The statistical significance of the indirect association was evaluated using 5,000 bootstrap resamples and 95% bias-corrected confidence intervals. All statistical analyses were conducted using IBM SPSS Statistics 27, and statistical significance was determined at the 0.05 level. Given the cross-sectional design, the resulting indirect effect was interpreted as a statistical indirect association rather than as evidence of a causal or temporal mediation process.

### 3.4 Data collection procedure

Data were collected through an online survey. Prior to data collection, all measurement instruments were integrated into a single questionnaire using Google Forms. The first section of the questionnaire included an informed consent statement explaining the purpose of the study, the voluntary nature of participation, and the confidentiality of participants’ responses. Participants were informed that the data would be used solely for scientific research purposes and that no personally identifiable information would be collected. The survey link was distributed to Turkish language teachers employed in public schools affiliated with the Ministry of National Education in Türkiye through various online communication channels. Participation was entirely voluntary, and respondents were permitted to complete the questionnaire only once. Data collection was conducted between April 14 and April 25, 2026, and responses were automatically recorded through the Google Forms platform. Following the completion of data collection, the dataset was screened for missing values, outliers, and inconsistent responses. The cleaned dataset was subsequently prepared for statistical analyses.

## 4 Result

When the descriptive statistics and correlation coefficients presented in Table 2 are considered together, the findings indicate that professional commitment (M = 50.43, SD = 7.25), psychological well-being (M = 45.48, SD = 5.74), and teacher self-efficacy (M = 37.10, SD = 7.75) satisfied the assumption of normality and were therefore considered suitable for subsequent analyses. Pearson correlation analyses revealed positive and statistically significant associations among all three study variables. Psychological well-being was positively associated with professional commitment (r = 0.35, *p* < 0.01), teacher self-efficacy was positively associated with professional commitment (r = 0.54, *p* < 0.01), and psychological well-being was positively associated with teacher self-efficacy (r = 0.49, *p* < 0.01).

**Table 2:** Descriptive statistics, correlations, and multicollinearity diagnostics of the study variables.

Variable	Descriptive Statistics						Correlations		
	Mean	SD	Skew	Kurt	Tol.	VIF	a	b	c
a: PC	50.43	7.25	−0.44	−0.13	—	—	1		
b: PWB	45.48	5.74	−0.85	0.87	0.76	1.31	0.35^**^	1	
c: TSE	37.10	7.75	−0.07	1.65	0.76	1.31	0.54^**^	0.49^**^	1

PC = professional commitment; PWB = psychological well-being; TSE = teacher self-efficacy; SD = standard deviation; Tol. = tolerance; VIF = variance inflation factor; ^**^p < 0.01.

Multicollinearity diagnostics further indicated that multicollinearity was not a concern. The tolerance value was 0.76 and the VIF value was 1.31 for both predictor variables. Taken together, these preliminary analyses supported proceeding with the proposed regression-based indirect-effect analysis.

Table 3 presents the results of the simple linear regression analysis conducted to examine the association between psychological well-being and professional commitment prior to testing the proposed indirect-effect model. The regression model was statistically significant, *F* = 46.87, *p* < 0.001. Psychological well-being accounted for approximately 13% of the variance in teachers’ professional commitment (R^2^ = 0.13).

**Table 3:** Regression analysis of the overall association between psychological well-being and professional commitment.

Predictor	Coeff.	SE	*p*	F	R^2^
Constant	32.54	2.62	<0.001		
PWB	0.39	0.06	<0.001	46.87	0.13

Coeff. = regression coefficient; SE = standard error; R^2^ = coefficient of determination.

Examination of the regression coefficients indicated a positive and statistically significant association between psychological well-being and professional commitment (B = 0.39, *p* < 0.001). This finding provides evidence of a significant association between the two variables prior to inclusion of these variables in the indirect-effect model. Accordingly, this result provides an empirical basis for examining the potential indirect association between psychological well-being and professional commitment through teacher self-efficacy.

The results presented in Table 4 reveal a consistent pattern of associations among psychological well-being, teacher self-efficacy, and professional commitment. First, psychological well-being was positively and significantly associated with teacher self-efficacy (B = 0.62, *p* < 0.001). This finding indicates that teachers reporting higher levels of psychological well-being also tended to report higher levels of perceived professional competence.

**Table 4:** The indirect association between psychological well-being and professional commitment through teacher self-efficacy.

Model	TSE (M)				PC (Y)		
	B	SE	t		B	SE	t
PWB (X) *a*	0.62	0.06	10.15	*c’*	0.13	0.06	2.23
TSE (M)	—	—	—	*b*	0.43	0.05	9.09
Constant	8.99	2.71	3.32		28.72	2.70	10.64
	R^2^ = 0.24, *F*_(1,328)_ = 103.01, *p* < 0.001				R^2^ = 0.30, *F*_(2,327)_ = 70.61, *p* < 0.001		
Bootstrap results for indirect effects			B		Boot SE	BootLLCI	BootULCI
Indirect Effect	TSE		0.26		0.04	0.19	0.36

PWB = psychological well-being; TSE = teacher self-efficacy; PC = professional commitment; X = independent variable; M = intervening variable; Y = dependent variable; B = unstandardized regression coefficient; SE = standard error; Boot SE = bootstrap standard error; BootLLCI = bootstrap lower-level confidence interval; BootULCI = bootstrap upper-level confidence interval; R^2^ = coefficient of determination.

Psychological well-being was also positively and significantly associated with professional commitment after teacher self-efficacy was included in the model (B = 0.13, *p* = 0.026). Although this association was comparatively smaller, it remained statistically significant, indicating that psychological well-being and professional commitment were significantly related even when teacher self-efficacy was taken into account.

Teacher self-efficacy was positively and significantly associated with professional commitment after psychological well-being was included in the regression model (B = 0.43, *p* < 0.001). This coefficient represents the unstandardized partial regression association between teacher self-efficacy and professional commitment, whereas the zero-order Pearson correlation between these variables was r = 0.54 (*p* < 0.01), as reported in Table 2. This result indicates that teachers who reported stronger beliefs in their ability to effectively fulfill their professional responsibilities also tended to report stronger commitment to their profession.

Bootstrap analyses further indicated a statistically significant indirect association between psychological well-being and professional commitment through teacher self-efficacy (B = 0.26, Boot SE = 0.04, 95% CI [0.19, 0.36]). Because the confidence interval did not include zero, the indirect association was statistically significant. Moreover, the association between psychological well-being and professional commitment remained statistically significant after teacher self-efficacy was included in the model. Given the cross-sectional nature of the data, this pattern should be interpreted as evidence of a statistically significant indirect association rather than as evidence of a causal process.

Regarding the explanatory power of the model, psychological well-being accounted for approximately 24% of the variance in teacher self-efficacy (R^2^ = 0.24). Furthermore, psychological well-being and teacher self-efficacy jointly accounted for approximately 30% of the variance in professional commitment (R^2^ = 0.30). Taken together, these findings indicate that the proposed model provides meaningful explanatory information regarding the statistical relationships among psychological well-being, teacher self-efficacy, and professional commitment.

Figure 1 illustrates the statistical relationships among psychological well-being, teacher self-efficacy, and professional commitment within the proposed indirect-effect model. Psychological well-being was positively associated with teacher self-efficacy, and teacher self-efficacy was positively associated with professional commitment. The indirect association between psychological well-being and professional commitment through teacher self-efficacy was statistically significant. In addition, the association between psychological well-being and professional commitment remained statistically significant after teacher self-efficacy was included in the model, although its magnitude was reduced. This pattern is consistent with the hypothesized indirect association. However, given the cross-sectional nature of the study, these findings should be interpreted as statistical associations and should not be taken as evidence of causal or temporal relationships among the variables.

**Figure 1:**
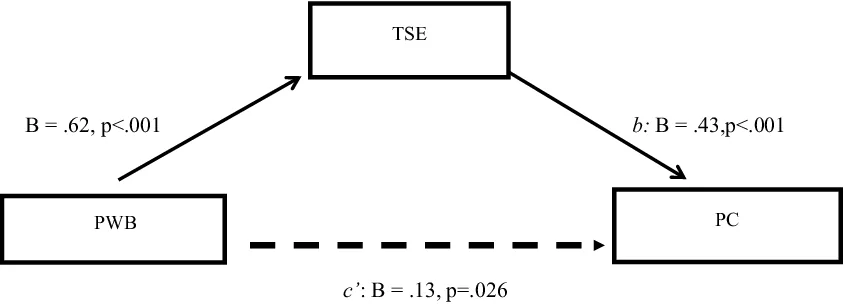
The indirect association between psychological well-being and professional commitment through teacher self-efficacy. PWB = psychological well-being; TSE = teacher self-efficacy; PC = professional commitment. B = unstandardized regression coefficient. The total association between psychological well-being and professional commitment was B = 0.39, *p* < 0.001 (c path). The direct association after accounting for teacher self-efficacy was B = 0.13, *p* = 0.026 (c′ path).

## 5 Discussion and conclusion

The present study examined the relationships among psychological well-being, teacher self-efficacy, and professional commitment within the framework of Social Cognitive Theory (Bandura, 2001), with a particular focus on the indirect association through teacher self-efficacy. Overall, the findings supported the proposed hypotheses and were largely consistent with both the theoretical assumptions and empirical evidence reported in the existing literature.

The first hypothesis, which proposed a positive association between psychological well-being and teacher self-efficacy, was supported. Psychological well-being was positively and significantly associated with teacher self-efficacy (B = 0.62, *p* < 0.001), accounting for approximately 24% of the variance in teacher self-efficacy (R^2^ = 0.24). This finding indicates that teachers reporting higher levels of psychological well-being also tended to report higher levels of perceived professional competence. The result is consistent with Social Cognitive Theory, which emphasizes the interrelationships among personal psychological resources, efficacy beliefs, and professional functioning (Bandura, 2001). From this theoretical perspective, psychological well-being and self-efficacy may represent interrelated psychological resources relevant to teachers’ capacity to cope with professional challenges and instructional demands. Importantly, given the cross-sectional nature of the present study, the observed association should not be interpreted as evidence that psychological well-being causes or temporally precedes teacher self-efficacy. Rather, the finding demonstrates a significant statistical association between these constructs and is consistent with previous studies reporting relationships between psychological well-being and self-efficacy in teaching and related professional contexts (Chen et al., 2024; Fırat, 2025; Shao, 2023; Xi-yun et al., 2022; Zhang et al., 2023).

The second hypothesis, which proposed a positive association between psychological well-being and professional commitment, was also supported. Psychological well-being was positively and significantly associated with professional commitment (B = 0.39, *p* < 0.001), accounting for approximately 13% of the variance in professional commitment (R^2^ = 0.13). This finding indicates that teachers reporting higher levels of psychological well-being also tended to report stronger professional commitment. The result is consistent with previous research reporting positive associations between psychological well-being and work-related outcomes such as commitment, engagement, and occupational persistence (Heidari et al., 2022; Kundi et al., 2020; Lee and Kim, 2023b). Furthermore, the association between psychological well-being and professional commitment remained statistically significant after teacher self-efficacy was included in the model (B = 0.13, *p* = 0.026), although its magnitude was reduced. This pattern is consistent with the possibility that teacher self-efficacy statistically accounts for part of the association between psychological well-being and professional commitment. However, because the data were collected at a single time point, the observed indirect pattern does not establish temporal ordering or causal direction among these variables.

The third hypothesis, which proposed a positive association between teacher self-efficacy and professional commitment, was supported. Teacher self-efficacy was positively and significantly associated with professional commitment after psychological well-being was taken into account (B = 0.43, *p* < 0.001). This finding indicates that teachers who reported stronger beliefs in their ability to effectively fulfill their professional responsibilities also tended to report stronger commitment to their profession. The finding is consistent with previous studies reporting positive associations between teacher self-efficacy and professional outcomes such as professional commitment, occupational persistence, and career sustainability (Mokhtar et al., 2023; Wang and Zhang, 2021; Yurtseven and Dulay, 2022; Zee and Koomen, 2016). Teachers with stronger efficacy beliefs may also report a greater capacity to cope with professional challenges and derive meaning from their work, characteristics that have been associated with sustained professional engagement in previous research (Klassen and Tze, 2014; Shu, 2022). Nevertheless, because the present study employed a cross-sectional design, this association should not be interpreted as evidence that teacher self-efficacy causally increases professional commitment.

The fourth hypothesis concerned the indirect association between psychological well-being and professional commitment through teacher self-efficacy. Bootstrap analyses indicated a statistically significant indirect association (B = 0.26, Boot SE = 0.04, 95% CI [0.19, 0.36]), thereby supporting H4. The association between psychological well-being and professional commitment also remained statistically significant after teacher self-efficacy was included in the model (B = 0.13, *p* = 0.026). Thus, the observed pattern is consistent with the theoretically proposed indirect relationship. This finding is compatible with Social Cognitive Theory, which emphasizes the interrelationships among psychological resources, efficacy beliefs, and professional functioning (Bandura, 2001). It is also broadly consistent with previous research reporting statistical relationships among self-efficacy, psychological well-being, and professional outcomes (Altınay and Bicentürk, 2023; Gan and Cheng, 2021; Li et al., 2024). Importantly, because the present data are cross-sectional, the statistically significant indirect association should not be interpreted as evidence that teacher self-efficacy causally transmits an effect of psychological well-being to professional commitment or as establishing the temporal sequence specified in the proposed model. Rather, the findings indicate that the observed covariance pattern is consistent with the theoretically proposed indirect association.

At the same time, the observed indirect association indicates that teacher self-efficacy accounts for only part of the statistical relationship between psychological well-being and professional commitment. This suggests that other psychological, organizational, and contextual factors may also be relevant to the relationship between these constructs. Variables such as job satisfaction, resilience, psychological capital, professional identity, perceived organizational support, and school climate may warrant consideration in future research. Examining these factors within longitudinal or multi-wave designs could provide a more comprehensive understanding of how psychological well-being, teacher self-efficacy, and professional commitment are related over time.

Taken together, the findings reveal a coherent pattern of statistically significant associations among psychological well-being, teacher self-efficacy, and professional commitment. Psychological well-being and teacher self-efficacy jointly accounted for approximately 30% of the variance in professional commitment (R^2^ = 0.30), indicating meaningful explanatory power within the proposed statistical model. The significant indirect association through teacher self-efficacy further suggests that self-efficacy may represent an important psychological correlate in understanding the relationship between psychological well-being and professional commitment. These findings should nevertheless be interpreted as associational rather than causal because of the cross-sectional nature of the study.

Overall, the present study provides an incremental and context-sensitive contribution to the literature by examining psychological well-being, teacher self-efficacy, and professional commitment within a single theoretically informed model among Turkish language teachers in Türkiye. Rather than demonstrating a novel causal mechanism, the findings provide context-specific evidence that teacher self-efficacy statistically accounts for part of the association between psychological well-being and professional commitment. This contribution is particularly relevant because the interrelationships among these constructs have received comparatively limited attention within this specific professional and educational context. Nevertheless, the cross-sectional nature of the data precludes conclusions regarding temporal ordering or causal relationships. Accordingly, the findings should be interpreted as theoretically meaningful statistical associations rather than as confirmation of a causal process.

## 6 Practical implications

The findings of this study offer several considerations for educational policymakers, school administrators, and teacher education programs. The observed associations indicate that psychological well-being and teacher self-efficacy may be relevant factors to consider in initiatives concerned with professional commitment and teacher retention. In particular, the significant associations of psychological well-being with both teacher self-efficacy and professional commitment suggest that organizational conditions supporting teachers’ psychological well-being warrant attention. However, given the cross-sectional nature of the study, these findings should not be interpreted as evidence that improving psychological well-being will necessarily lead to increased self-efficacy or professional commitment.

Given that psychological well-being was significantly associated with both teacher self-efficacy and professional commitment, initiatives aimed at supporting teachers’ emotional resilience and subjective well-being may warrant consideration and further empirical evaluation. Professional development programs may consider incorporating psychological competencies such as self-regulation, resilience, and stress-management strategies alongside pedagogical knowledge and skills (Chen et al., 2024; Zee and Koomen, 2016). In addition, mentoring systems, peer-support networks, and school-based counseling services represent potential forms of organizational support whose contribution to teachers’ psychological well-being could be examined in future intervention-based research.

The finding that teacher self-efficacy was significantly associated with professional commitment and was involved in a statistically significant indirect association highlights the potential relevance of self-efficacy in understanding teachers’ professional commitment. Accordingly, pre-service and in-service teacher education programs may consider providing opportunities related to the primary sources of self-efficacy identified by Bandura (1997), including mastery experiences, vicarious learning, verbal persuasion, and positive emotional experiences. For novice teachers, structured mentoring and opportunities for reflective practice in authentic classroom settings may provide relevant contexts for supporting self-efficacy beliefs. For experienced teachers, continuous professional development and opportunities to share effective practices may similarly warrant consideration. The potential effectiveness of such approaches, however, should be evaluated through longitudinal and intervention-based research.

More broadly, the findings may inform further consideration of teacher well-being within educational policy and organizational practice. Professional commitment is likely to be associated not only with individual psychological factors but also with broader systemic and organizational conditions, including school climate, workload, opportunities for professional growth, and participation in decision-making. Accordingly, psychologically supportive school environments, work–life balance, equitable workload practices, and opportunities for teachers to participate in institutional decision-making may warrant consideration in broader discussions of teacher retention and professional sustainability. Future research should examine how these organizational factors interact with psychological well-being and self-efficacy in relation to professional commitment.

Taken together, the findings suggest that psychological well-being and teacher self-efficacy are relevant psychological factors to consider in research and practice concerned with professional commitment and teacher sustainability. Although initiatives addressing these psychological resources may warrant further consideration, the present findings do not establish that such initiatives will increase professional commitment or improve broader educational outcomes. Accordingly, longitudinal and intervention-based studies are needed to evaluate whether and under what conditions changes in psychological well-being and self-efficacy are associated with subsequent changes in professional commitment.

## 7 Limitations and future directions

Several limitations should be considered when interpreting the findings of this study. First, the study employed a cross-sectional design, with all variables measured at a single time point. Consequently, temporal ordering and causal relationships among psychological well-being, teacher self-efficacy, and professional commitment cannot be established. In particular, the statistically significant indirect association identified in the present study should not be interpreted as evidence that psychological well-being causes changes in teacher self-efficacy or that teacher self-efficacy causally transmits an effect of psychological well-being to professional commitment. Rather, the indirect-effect analysis indicates that the observed pattern of associations is statistically consistent with the theoretically proposed model. Moreover, alternative or statistically equivalent models involving different directional orderings of these variables may also be compatible with the observed cross-sectional covariance structure. For example, stronger teacher self-efficacy may be associated with higher psychological well-being rather than psychological well-being necessarily preceding self-efficacy, and reciprocal relationships among these constructs are also theoretically plausible. Therefore, the ordering specified in the present model should be understood as theoretically informed rather than empirically established by the cross-sectional data. Longitudinal, cross-lagged, and experimental designs are needed to examine temporal ordering, reciprocal relationships, and potential causal processes among these constructs.

Second, the sampling strategy and representativeness of the sample constitute an important limitation. Participants were recruited through convenience sampling and voluntary participation using online communication channels. Consequently, the sample cannot be assumed to be statistically representative of the broader population of Turkish language teachers employed in public schools in Türkiye. The voluntary nature of participation also introduces the possibility of self-selection bias, as teachers who chose to respond may systematically differ from those who did not participate in terms of psychological well-being, self-efficacy, professional commitment, or other unmeasured characteristics. Moreover, the sample was not systematically compared with national population parameters; therefore, population-level generalizations cannot be made from the present data. Future studies should employ probability-based or more systematically stratified sampling strategies and, where possible, compare sample characteristics with national teacher demographics to strengthen external validity.

Additionally, an *a priori* power analysis was not conducted before data collection. Because no theoretically predetermined minimum effect size was specified prospectively, the required sample size was not established on an *a priori* basis. Future studies should determine sample-size requirements prospectively using theoretically meaningful expected effect sizes and appropriate power criteria.

A further limitation concerns the exclusive reliance on self-report measures collected within a single survey administration, which may introduce common method variance, social desirability effects, and other forms of systematic measurement error. Although Harman’s single-factor test indicated that the first factor accounted for only 30.20% of the total variance and therefore did not suggest a dominant common method factor, this test has limited sensitivity and cannot rule out common method bias entirely. Accordingly, the observed associations and indirect effect may be partly inflated by shared method variance arising from the use of self-report measures administered within the same survey, and their magnitudes should therefore be interpreted with appropriate caution. Future studies should incorporate procedural remedies and multiple data sources, such as classroom observations, peer assessments, performance evaluations, or administrative records, and may employ more rigorous statistical approaches to assess common method variance.

An additional measurement-related limitation concerns the respecification of the measurement models in the current sample. Although the respecified CFA models demonstrated acceptable fit for all three measures, several residual covariances were freely estimated on the basis of localized model misfit and substantive overlap in item content. These modifications were theoretically defensible and resulted in improved model fit; however, because they were identified using the present sample, they may partly reflect sample-specific characteristics. Accordingly, the respecified measurement structures should be cross-validated in independent and more diverse teacher samples before stronger conclusions are drawn regarding their generalizability. Another measurement-related limitation concerns the use of an aggregate professional commitment score in the primary analyses. Although the three dimensions of Professional Satisfaction, Social Gain, and Professional Career were moderately to strongly correlated and showed substantial loadings on a broader professional commitment factor, analyzing only the aggregate score may obscure dimension-specific patterns of association with psychological well-being and teacher self-efficacy. Future studies should therefore examine these dimensions separately to determine whether the observed relationships differ across specific aspects of professional commitment.

Furthermore, several variables identified in the literature as important predictors of professional commitment—including school climate, leadership practices, occupational stress, and emotional exhaustion—were not included in the proposed model. Consequently, a portion of the variance in professional commitment may be attributable to factors not examined in the present study, and the findings should therefore be interpreted with appropriate caution.

Finally, although a statistically significant indirect association through teacher self-efficacy was observed, this finding does not imply that self-efficacy is the only variable relevant to the relationship between psychological well-being and professional commitment. Future research may examine additional intervening or moderating variables, such as job satisfaction, emotional intelligence, resilience, psychological capital, and professional identity. The potential moderating roles of demographic and professional characteristics, including gender, teaching experience, and educational attainment, also warrant further investigation. Longitudinal and multi-wave designs would be particularly valuable for examining the temporal ordering of these relationships and for determining whether the observed indirect association is replicated over time.

## Data Availability

The raw data supporting the conclusions of this article will be made available by the authors, without undue reservation.
